# Inactivation of the Response Regulator AgrA Has a Pleiotropic Effect on Biofilm Formation, Pathogenesis and Stress Response in Staphylococcus lugdunensis

**DOI:** 10.1128/spectrum.01598-21

**Published:** 2022-02-09

**Authors:** Marion Aubourg, Marine Pottier, Albertine Léon, Benoit Bernay, Anne Dhalluin, Margherita Cacaci, Riccardo Torelli, Pierre Ledormand, Cecilia Martini, Maurizio Sanguinetti, Michel Auzou, François Gravey, Jean-Christophe Giard

**Affiliations:** a Université de Caen Normandie, Dynamicure, INSERM U1311, CHU de Caen, Caen, France; b LABÉO Frank Duncombe, Caen, France; c Plateforme Proteogen SFR ICORE 4206, Université de Caen Normandie, Caen, France; d Institute of Microbiology, Catholic University of Sacred Heart, L. go F. Vito 1, Rome, Italy; e Université de Caen Normandie, ABTE, Caen, France; f CHU de Caen, Laboratoire de Microbiologie, Caen, France; University of Calgary

**Keywords:** *Staphylococcus lugdunensis*, AgrA, transcriptional regulation, *agr* system

## Abstract

Staphylococcus lugdunensis is a coagulase-negative Staphylococcus that emerges as an important opportunistic pathogen. However, little is known about the regulation underlying the transition from commensal to virulent state. Based on knowledge of S. aureus virulence, we suspected that the *agr* quorum sensing system may be an important determinant for the pathogenicity of *S. lugdunensis*. We investigated the functions of the transcriptional regulator AgrA using the *agrA* deletion mutant. AgrA played a role in cell pigmentation: Δ*argA* mutant colonies were white while the parental strains were slightly yellow. Compared with the wild-type strain, the Δ*argA* mutant was affected in its ability to form biofilm and was less able to survive in mice macrophages. Moreover, the growth of Δ*agrA* was significantly reduced by the addition of 10% NaCl or 0.4 mM H_2_O_2_ and its survival after 2 h in the presence of 1 mM H_2_O_2_ was more than 10-fold reduced. To explore the mechanisms involved beyond these phenotypes, the Δ*agrA* proteome and transcriptome were characterized by mass spectrometry and RNA-Seq. We found that AgrA controlled several virulence factors as well as stress-response factors, which are well correlated with the reduced resistance of the Δ*agrA* mutant to osmotic and oxidative stresses. These results were not the consequence of the deregulation of RNAIII of the *agr* system, since no phenotype or alteration of the proteomic profile has been observed for the ΔRNAIII mutant. Altogether, our results highlighted that the AgrA regulator of *S. lugdunensis* played a key role in its ability to become pathogenic.

**IMPORTANCE** Although belonging to the natural human skin flora, Staphylococcus lugdunensis is recognized as a particularly aggressive and destructive pathogen. This study aimed to characterize the role of the response regulator AgrA, which is a component of the quorum-sensing *agr* system and known to be a major element in the regulation of pathogenicity and biofilm formation in Staphylococcus aureus. In the present study, we showed that, contrary to S. aureus, the *agrA* deletion mutant produced less biofilm. Inactivation of *agrA* conferred a white colony phenotype and impacted *S. lugdunensis* in its ability to survive in mice macrophages and to cope with osmotic and oxidative stresses. By global proteomic and transcriptomic approaches, we identified the AgrA regulon, bringing molecular bases underlying the observed phenotypes. Together, our data showed the importance of AgrA in the opportunistic pathogenic behavior of *S. lugdunensis* allowing it to be considered as an interesting therapeutic target.

## INTRODUCTION

Thanks to gene regulatory networks, pathogenic bacteria can rapidly adapt to their environment and modulate the expression of virulence-associated factors. First described in Staphylococcus aureus, the *agr* locus (for accessory gene regulator) combines a classical two-component system and a quorum-sensing system (1, 2). In addition, it plays a role in the regulation of virulence factors and metabolism genes (3, 4). This *agr* locus comprises two divergent transcripts, RNAII and RNAIII, driven by the P2 and P3 promoters, respectively (5). RNAII is an operon that encodes four genes (*agrBDCA*), within *agrB* and *agrD* genes are involved in quorum-sensing while *agrC* and *agrA* genes constitute the two-component system. The *agr* system appears conserved throughout the Staphylococcus genus, but sequence variations are mainly present in the *agrB*, *agrD*, and *agrC* genes whereas the *agrA* sequence is much more conserved (6). AgrD is the precursor of the auto-inducing peptide (AIP) which is secreted by the membrane protein AgrB. The accumulation of AIP in the extracellular matrix is detected by the transmembrane histidine kinase AgrC, which in turn induces the activation of the transcriptional regulator AgrA by phosphorylation (5, 7). Phosphorylated AgrA, via its C-terminal LytTR domain, recognizes imperfect direct repeats of DNA with a consensus sequence (TA)(AC)(CA)GTTN(AG)(TG) located in the intergenic region between the P2 and P3 promoters (8). In addition to the regulation of its own promoter (P2) and RNAIII, AgrA directly regulates transcription of genes encoding phenol-soluble modulin (PSM) peptides as well as *bsaA* encoding the glutathione peroxidase, essential for S. aureus resistance to oxidative stress (9, 10). A study on gene expression also showed the important role of the *agr* locus in the control of catabolic pathways, nutrient uptake, and energy metabolism (4). RNAIII is a pleiotropic effector that upregulates transcription of most extracellular protein genes and downregulates the transcription of many surface protein genes (7, 11). In S. aureus, the RNAIII transcript contains the *hld* gene encoding the delta-hemolysin which is an important virulence factor of S. aureus (7, 12).

*S. lugdunensis* is a coagulase-negative staphylococci (CoNS) that is part of the commensal human skin flora but is distinguished from other CoNS species due to its ability, as S. aureus, to cause many infections such as acute endocarditis, skin and soft tissue infections, pneumonia, meningitis and osteoarticular infections (13, 14). It is probably the most aggressive CoNS species and several virulence factors have been characterized (15). Likewise, S. lugdunensis is well-equipped for the acquisition of nutritional iron, which has been shown to be crucial for biofilm formation, virulence, and resistance to oxidative stress (16, 17). This *a priori* harmless commensal CoNS should therefore be regarded as an opportunistic pathogen. In this context, the regulation of immune system evasion and stress response mechanisms orchestrated by transcriptional regulators constitutes the keys to explain the ambivalence of this species. LytSR is the only regulatory system that has been experimentally studied in S. lugdunensis (18). This two-component system appeared implicated in pathogenesis likely due to its involvement in biofilm formation and the control of the transcription of virulence factors such as fibrinogen-binding protein (Fbl), autolysin (Atl), and the type VII secretion system (18). The *agr* locus of S. lugdunensis has a genomic organization similar to that of S. aureus, with 63% sequence homology, and with a highly conserved intergenic region comprising the P2 and P3 promoters (19). Interestingly, the *hld* gene is in the RNAIII transcript in most staphylococci except S. lugdunensis and S. saprophyticus (20, 21). Therefore, the synergistic hemolytic activity is generated by three peptides encoded by the *slushABC* locus in *S. lugdunensis* (22). However, the absence of the *hld* gene did not alter the ability of S. lugdunensis RNAIII to function as a regulatory molecule since it was able to induce transcriptional and phenotypic complementation of several *agr*-regulated exoproteins in an S. aureus *agr* mutant (23).

Based on these data, the present study aimed to investigate the role of the transcriptional regulator ArgA in S. lugdunensis. We constructed the Δ*agrA* deletion strain of S. lugdunensis N920143 and showed that this mutation had an impact on cell pigmentation, biofilm formation, oxidative and osmotic stress responses, and survival in mice macrophages. In addition, to identify the AgrA regulon and molecular determinants underlying these phenotypes, we performed a comprehensive proteomic study and genome-wide analysis by mass spectrometry and RNA-seq, respectively.

## RESULTS

### Phenotypic characterization of S. lugdunensis N920143 Δ*agrA* mutant.

**FIG 1:**
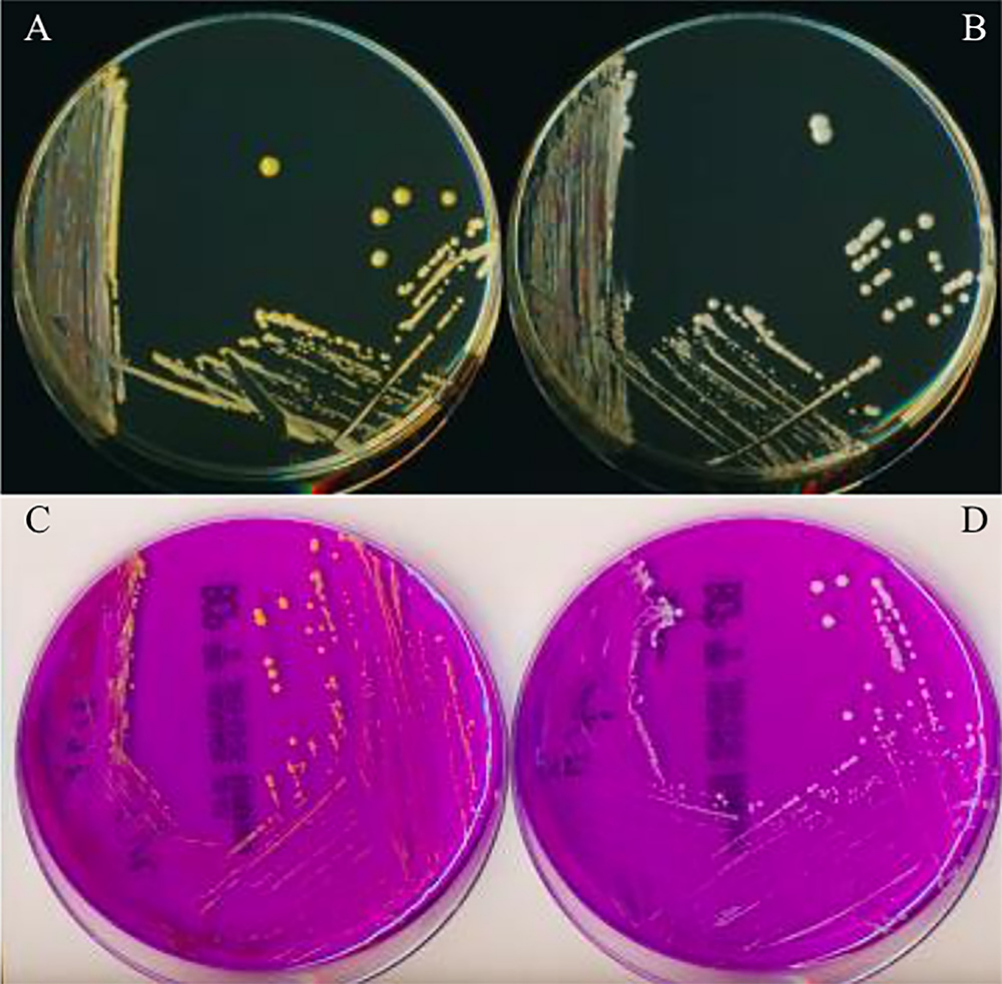
Pigmentation of the bacterial colonies of S. lugdunensis N920143 and Δ*agrA* mutant strains. A–C: Yellow colonies of the S. lugdunensis N920143 strain after 24 h of incubation on TS (A) and BCP (C) agar. B–D: White colonies of the Δ*agrA* mutant after 24 h of incubation on TS (B) and BCP (D) agar.

To investigate the role of AgrA of S. lugdunensis, the corresponding deletion mutant was constructed and phenotypically analyzed. First, Δ*agrA* mutant and its parental strains displayed the same growth curves under standard condition at 37°C in Brain Heart Infusion (BHI) as well as the same antimicrobial susceptibility profiles (data not shown). In addition, transmission electron microscopy of cells harvested at the onset of stationary phase and after 24 h did not reveal any morphological difference between wild type (WT) and mutant strains (data not shown). S. lugdunensis N920143 naturally synthetizes slight yellow pigment which macroscopically colors the colonies, and surprisingly, the Δ*agrA* mutant colonies appeared clearly whiter on Tryptic Soy (TS) (Fig. 1A and B) as well as on BromoCresol Purple (BCP) agar (Fig. 1C and D).

**FIG 2:**
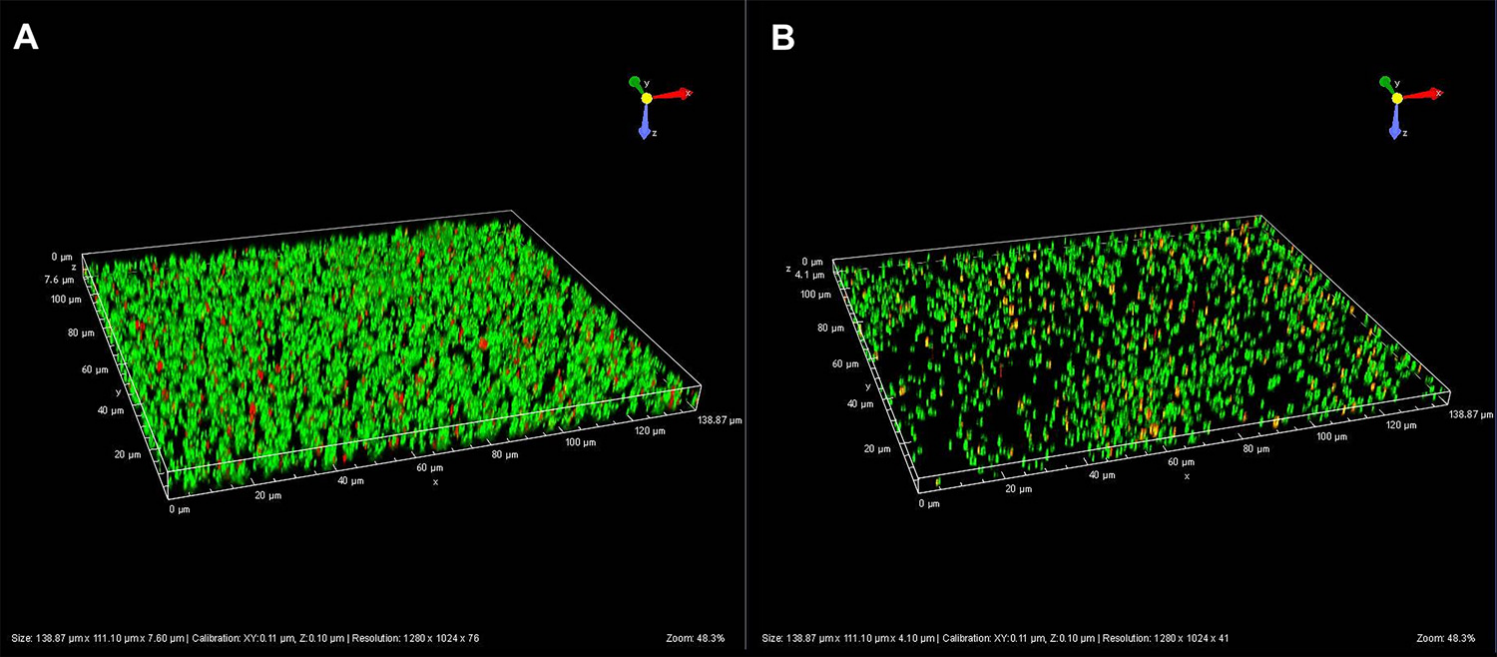
Fluorescence microscopy images of S. lugdunensis N920143 (A) and Δ*agrA* mutant (B) which generated biofilms after 20 h of growth in BHI.

**FIG 3:**
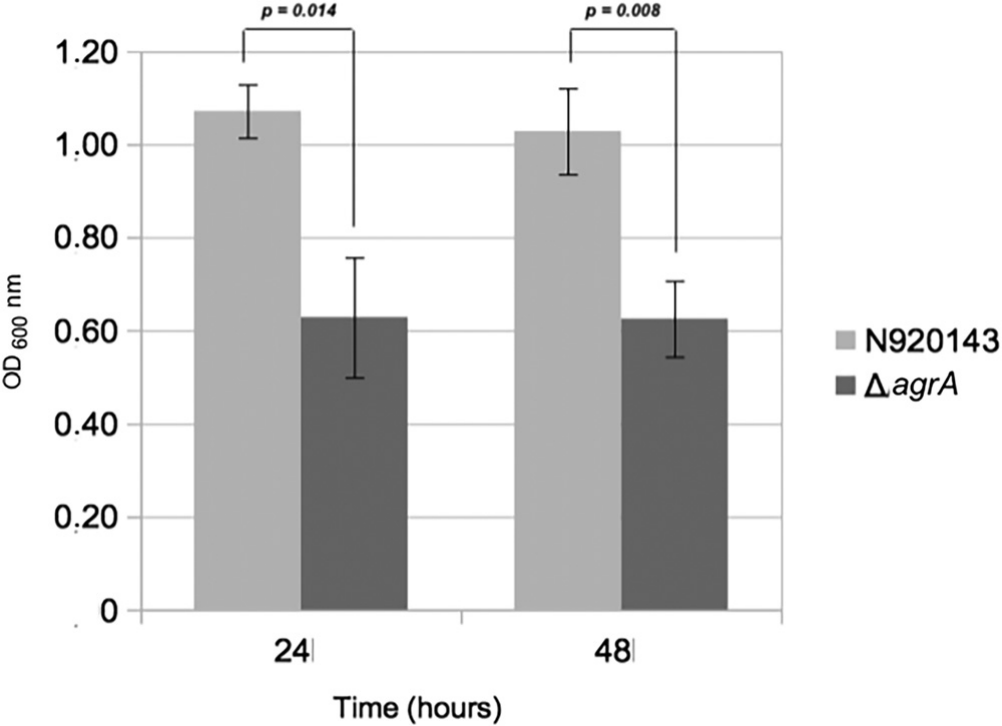
Biofilm formation by S. lugdunensis N920143 (gray) and Δ*agrA* mutant (dark gray) strains after 24 h and 48 h of growth in BHI. Error bars represent the standard deviations of three independent experiments.

Because the quorum-sensing system contributes to the synchronization of mechanisms leading to biofilm formation (2, 3), we analyzed the role of the AgrA regulator in this phenotype. By 3D-microscopy technology, we observed that the biofilm produced by the WT strain was obviously denser (Fig. 2A) than that produced by the Δ*agrA* mutant of S. lugdunensis (Fig. 2B). Based on three independent experiments, means quantifications of the biofilm volumes were 16,707 µm^3^ and 2,402 µm^3^ for the WT and the mutant strains, respectively (*P = *0.037). To confirm the reduced ability of the Δ*agrA* mutant to produce biofilm, we showed that, after one or 2 days in BHI, the Δ*agrA* mutant produced half less biofilm in polypropylene microtiter plates than its parental strain by measuring crystal violet staining (Fig. 3).

**FIG 4:**
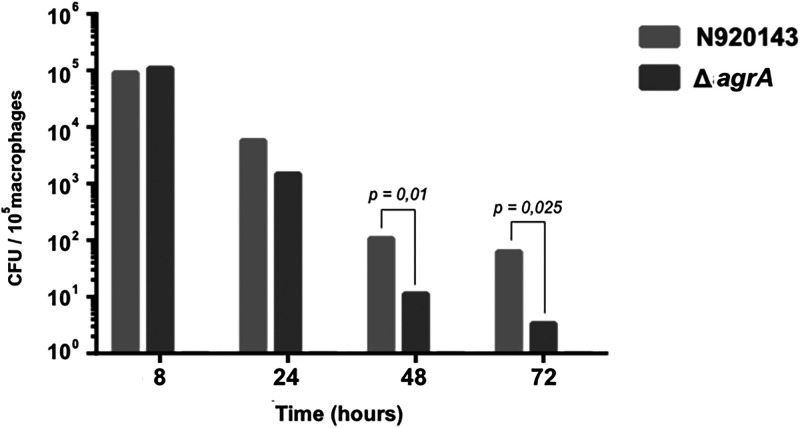
Time course of intracellular survival of S. lugdunensis N920143 (gray) and Δ*agrA* mutant (dark gray) within mice peritoneal macrophages. The results are numbers of viable intracellular bacteria per 10^5^ macrophages and are the means ± standard deviations from three independent experiments with three wells each.

To know if AgrA may be a virulence factor in S. lugdunensis, survival of S. lugdunensis N920143 and Δ*agrA* mutant cells in murine peritoneal macrophages was carried out. Over 3 days, significantly fewer Δ*agrA* mutant cells were retrieved into mice macrophages than S. lugdunensis N920143 cells. As shown in Fig. 4 1.1 × 10^1^ and 0.33 × 10^1^ CFU (colony forming units) of mutant cells were enumerated into 10^5^ macrophages after 48 h and 72 h, respectively, whereas 1 × 10^2^ and 6.2 × 10^1^ CFU of *S. lugdunensis* N920143 were counted (*P = *0.01, *P = *0.025, respectively). These data strongly argued for an important role of AgrA in the pathogenicity of S. lugdunensis.

**FIG 5:**
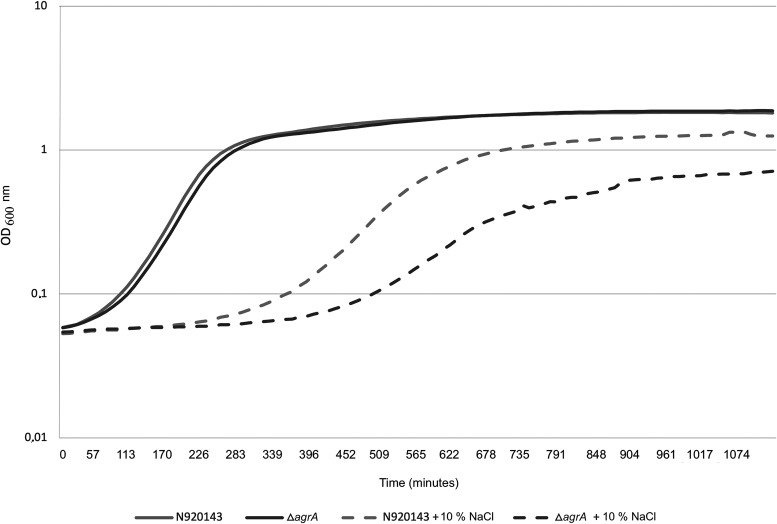
Representative growth curves of S. lugdunensis N920143 in BHI (continuous gray line), S. lugdunensis N920143 in BHI with 10% of NaCl (spaced gray line), Δ*agrA* mutant in BHI (continuous dark gray line), and Δ*agrA* mutant in BHI with 10% of NaCl (spaced dark gray line).

Next, in order to show the role of AgrA in the bacterial fitness, we evaluated whether AgrA of S. lugdunensis was involved in mechanisms of resistance to osmotic stress. In the presence of 10% NaCl, the growth of the Δ*agrA* mutant cells was much more impacted than that of the WT: 825 min and 525 min were required to reach an OD_600_ (optical density measured at 600 nm) of 0.5 for the Δ*agrA* mutant and parental cells, respectively (Fig. 5). Note that at the same OD_600_, the number of CFU was similar for both strains and corresponded to 17.3 ± 5 × 10^7^ CFU/ml when the OD_600_ reached 0.5. Moreover, in stationary phase, the OD_600_ under the osmotic stress condition was reduced for the mutant strain (0.7 versus 1.25 for the WT, *P = *0.0012) indicating a lower cell density (Fig. 5).

**FIG 6:**
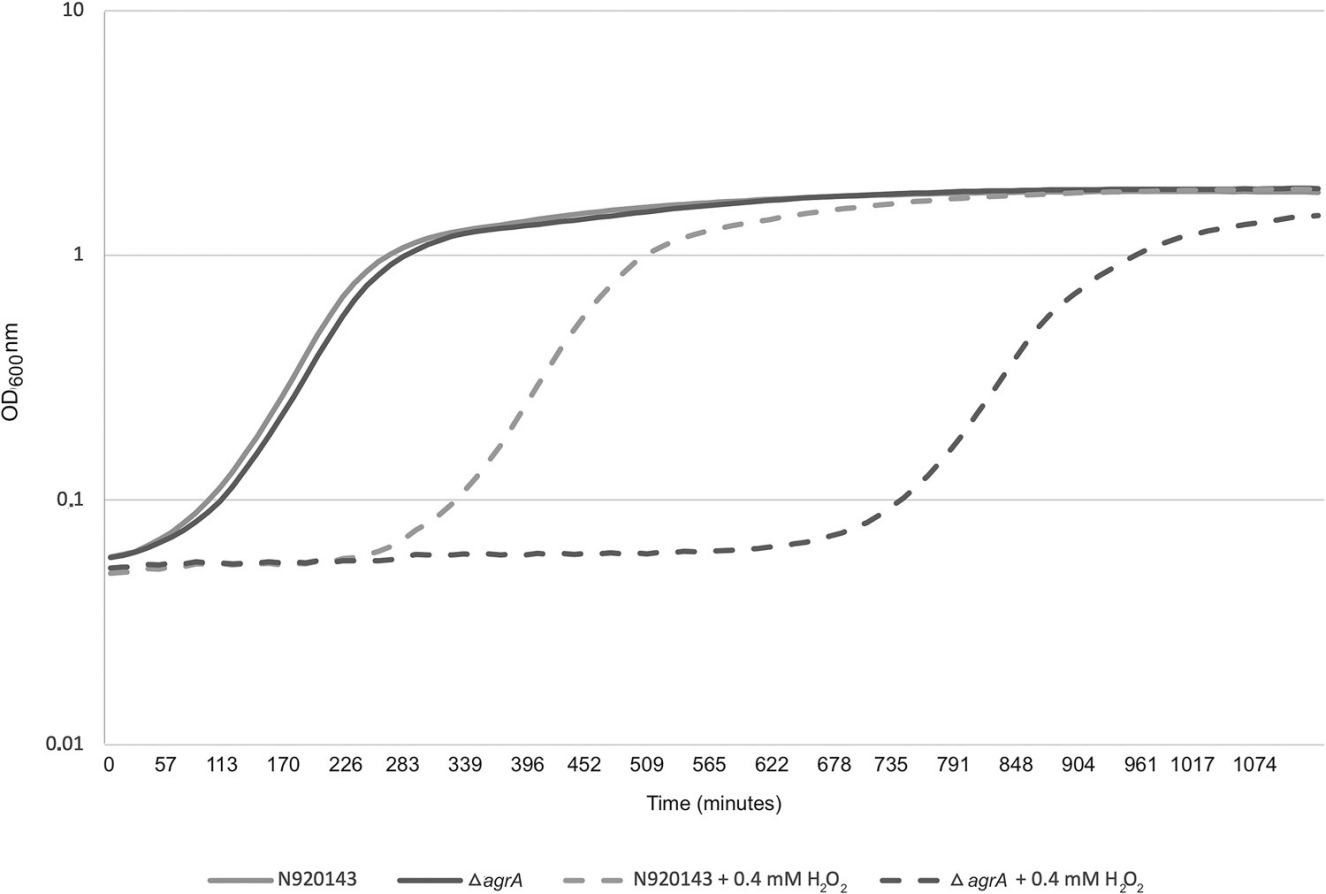
Representative growth curves of S. lugdunensis N920143 in BHI (continuous gray line), *S. lugdunensis* N920143 in BHI with 0.4 mM H_2_O_2_ (spaced gray line), Δ*agrA* mutant in BHI (continuous dark gray line), and Δ*agrA* mutant in BHI with 0.4 mM H_2_O_2_ (spaced dark gray line).

**FIG 7:**
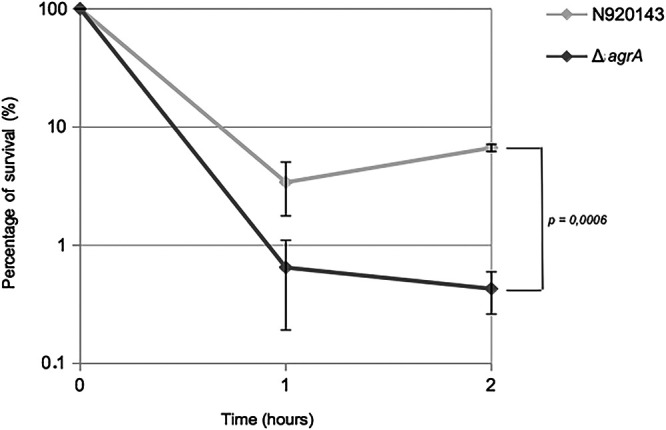
Representative survival curves of S. lugdunensis N920143 in BHI with 1 mM H_2_O_2_ (gray) and Δ*agrA* mutant in BHI with 1 mM H_2_O_2_ (dark gray). Mean, SD, and *P value* are presented.

The role of AgrA in the ability of S. lugdunensis to cope with reactive oxygen species (ROS) was then studied. As observed in Fig. 6, the increased lag phase of growth triggered by the addition of 0.4 mM H_2_O_2_ was much more pronounced for the Δ*agrA* mutant than for the WT strain. Under this oxidative stress condition, OD_600_ of 0.5 was reached after around 7 h and more than 14 h for S. lugdunensis N920143 and Δ*agrA* mutant, respectively. We also confirmed that AgrA conferred resistance to oxidative stress by comparison of survival of S. lugdunensis N920143 and Δ*agrA* mutant cells in the presence of a lethal concentration of H_2_O_2_ (Fig. 7). After 2 h with 1 mM H_2_O_2_, the rates of survival of S. lugdunensis N920143 and Δ*agrA* mutant were 6.7% and 0.4%, respectively (*P = *0.0006) (Fig. 7). Our data demonstrated that AgrA of S. lugdunensis was involved in oxidative stress response.

To verify that Δ*agrA* mutant phenotypes observed were exclusively due to the lack of *agrA*, we complemented the mutant by transforming an intact copy of *the agr* operon with its promoter cloned into the replicative vector pRB473. Compared with the Δ*agrA* mutant transformed with the empty pRB473 plasmid, this complementation made it possible to restore better growth in the presence of 10% NaCl allowing reaching an OD_600_ of 1.33 (versus 1.05 for the control strain) (Fig. S1A). Likewise, the lag phase was reduced by 2 h for the complemented strain when it was cultured with 0.4 mM H_2_O_2_ (Fig. S1B).

### The S. lugdunensis *agrA* proteome.

**FIG 8:**
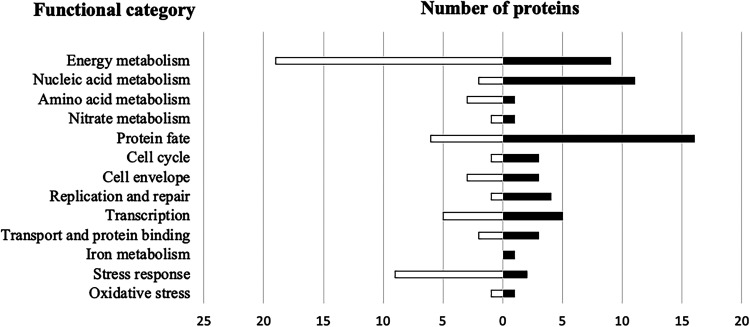
Functional classification of proteins significantly upregulated (black bars) and downregulated (white bars) of growing cell (OD_600_ of 1) of S. lugdunensis N920143 compared to Δ*agrA* mutant.

The S. lugdunensis *agrA* proteome was analyzed by mass-spectrometry after trypsin digestion. Among the 2368 proteins potentially encoded by the genome, 162 polypeptides appeared differentially expressed in the Δ*agrA* mutant regarding the following parameters: fold changes greater or smaller than 2 fold with a *P value* less than 0.05. 78 were significantly more produced while 84 were less abundant (Table S1, Fig. S2). The functional annotation of differentially produced proteins revealed various cellular metabolisms impacted by the deletion of *agrA*: energy (*n* = 28), nucleic acids (*n* = 13), amino acids (*n* = 4), nitrate (*n* = 2), protein fate (*n* = 22) (Fig. 8). In addition, the inactivation of the transcriptional regulator AgrA also interfered with the expression of stress proteins. Interestingly, the autolysin, ferrous iron transport protein B and cold shock protein CspC were among the most over-produced proteins in the mutant strain. Conversely, enzymes involved in oxidative stress such as MsrA, MsrB (peptide methionine sulfoxide reductases), KatA (catalase), or in general stress response (Asp23, AmaP, Clp proteases, OpuC, general stress protein) were less abundant in the Δ*agrA* cells (Table S1, Fig. 8). Note that some of the corresponding genes were found significantly deregulated in our transcriptomic results (Table S2, see below).

### Global regulation of gene expression by AgrA.

**FIG 9:**
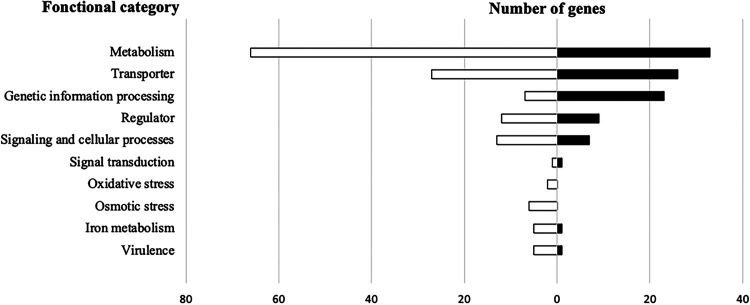
Schematic representation of the functional classification of genes upregulated (black bars) and downregulated (white bars) of S. lugdunensis N920143 cells compared to Δ*agrA* mutant cells (OD_600_ of 1).

To identify genes with altered expression due to the deletion of the transcriptional regulator AgrA, a global transcriptomic study of S. lugdunensis N920143 was undertaken and compared to Δ*agrA* mutant cells. Using S. lugdunensis N920143 as a reference genome, 98.46–99.61% of the high-quality sequenced reads (Phred score > 30) were uniquely mapped (Table S3). Overall, the Δ*agrA* deletion significantly affected the expression of 400 genes (Log_2_ fold change (Log_2_FC) less than -1 or higher than 1). Among them, 149 were upregulated and 251 were downregulated (Table S3, Fig. S3). Most upregulated genes included those involved in metabolism (*n* = 33), transport (*n* = 26) and genetic information processes (*n* = 23) whose 9 regulators (Fig. 9). Some downregulated genes were also associated with metabolism (*n* = 66), transport (*n* = 27), CRISPR system (*n* = 8) and transcriptional regulation (*n* = 11) (Fig. 9). Nonetheless, *agr* showed an important influence on the regulation of genes involved in the bacterial stress response and the virulence of S. lugdunensis (see below). With the aim to identify putative direct AgrA targets, we looked for the presence of the possible AgrA-binding box (WMMGTTNRK) in the promoter regions of the deregulated genes identified by our global transcriptomic study (8). This motif was recovered in front of 48 open reading frames which could constitute the direct ArgA regulon (Table S2).

As expected, transcription of RNAIII was dramatically reduced in the Δ*agrA* mutant (Table S2). It was thus tempting to hypothesize that phenotypes observed in the Δ*agrA* mutant could result from this deregulation of RNAIII. Therefore, we have constructed and analyzed the ΔRNAIII mutant strain of S. lugdunensis. Until yet, no phenotypical difference nor proteomic profile modification has been observed when compared with the WT strain. RT-qPCR performed with some genes identified by RNA-seq (*slushABC*, *crtM*, *crtN*, *katA*, *agrA* and RNAIII) confirmed the downregulation in the Δ*agrA* mutant as well as the absence of the RNAIII involvement (Table 1).

**TABLE 1:** Analysis of transcriptional level of *slushABC*, *crtM*, *crtN*, *katA*, *agrA*, and RNAIII genes in *S. lugdunensis* N920143 *versus* Δ*agrA* mutant or ΔRNAIII mutant

Gene	Fonction	*ΔagrA* mutant		ΔRNAIII mutant	
		Fold change	*P value*	Fold change	*P value*
*slushABC*	Hemolytic activity	−15.8	7.5e-05	1.2	0.57
*crtM*	Staphyloxanthin biosysnthesis	−12.4	2.8e-05	1.2	0.55
*crtN*	Staphyloxanthin biosysnthesis	−13.6	4.6e-05	1.1	0.73
*katA*	Oxydative stress	−5.1	1.0e-04	1.4	0.38
*agrA*	Regulator			1.2	0.93
RNAIII	Regulator	−88.5	0.84		

Note that the reduced expressions of RNAIII, *crtM*, *crtN*, *slushABC* and *SLUG_07290* (among the most deregulated genes observed by RNA-seq) in the Δ*agrA* mutant were significantly complemented with the addition of the pRB473-*agr* construction, especially for RNAIII and *slushABC* genes (Fig. S1C).

### Regulation of stress factors.

One of the most striking observations was the impact of *agrA* on the stress response, especially the oxidative and osmotic stresses. The transcription of three major genes involved in the detoxification of ROS, *katA* (encoding catalase), *sodA* (encoding superoxide dismutase) and SLU_18820 (encoding coenzyme A-disulfide reductase, CoADR) were significantly downregulated in the Δ*agrA* mutant (Table S2). It is noteworthy that KatA and CoADR were also less abundant in our proteomic study (Table S1). S. lugdunensis was obviously able to produce pigments and possesses *crtM* and *crtN* genes (likely in operon structure with SLUG_01810-01840 loci) coding for dehydrosqualene synthase and dehydrosqualene desaturase, respectively. CrtMN enzymes catalyzed the two first reactions of the pathway of staphyloxanthin biosynthesis in S. aureus (24). CrtM catalyzes the head-to-head condensation of two molecules of farnesyl diphosphate to form dehydrosqualene which is then dehydrogenated by CrtN, allowing the formation of 4,4’-diaponeurosporene that is a yellow carotenoid pigment (25). Using a *crtM* mutant of S. aureus, Clauditz and coworkers showed that such a biological antioxidant plays a role in fitness, resistance to human neutrophils and to oxidative stress by ROS scavenging (26). The downregulation of *crtMN* operon in the Δ*agrA* mutant of S. lugdunensis was well correlated with the reduced resistance to H_2_O_2_ in addition to the white color phenotype of the mutant.

Our transcriptomic results indicated that AgrA negatively regulated the expression of genes of synthesis of betaine from choline (*cudTAB* operon) and positively those involved in the osmoprotectant (glycine betaine) transport (*opuCABCD* operon) (Table S2). Staphylococci are of the most halotolerant eubacteria and face high NaCl concentrations by accumulating compatible solutes as glycine betaine (27). Based on our data, the Agr system seemed implicated in the osmotic stress resistance of S. lugdunensis by favoring import of osmoprotectants.

S. lugdunensis is particularly well equipped with iron-metabolism enzymes playing an important role in the “nutritional immunity” setting up by the host to limit access to essential ions. Interestingly, we identified several AgrA upregulated genes known to be involved in iron homeostasis (*isdB*, *isdE*, *efeB*) and hemin detoxification (SLUG_06770, SLUG_06780) (17).

Among stress-inducible genes members of the AgrA regulon, two genes coding for Clp ATPases (*clpB* and *clpL*) were under-expressed in the Δ*agrA* mutant (Table S2). ClpB and ClpL are molecular chaperones that are required for the development of induced thermotolerance in S. aureus (28). Of note that the ClpL protein was also recovered as less abundant in the mutant strain (Table S2).

### Regulation of the virulence genes.

The *agr* system with RNAIII are classically associated with the virulence of S. aureus since upregulate the production of degradative enzymes and toxins and downregulate proteins involved in colonization (11). In S. lugdunensis, the expression of some putative virulence factors appeared under AgrA control. *isaA*, encoding the immunodominant S. aureus antigen A (IsaA), was significantly overexpressed in the Δ*agrA* mutant of S. lugdunensis (Table S2). This lytic transglycosylase is both exposed to the cell surface and secreted and plays a role in peptidoglycan turnover, cell wall hydrolysis and cell separation (29). Moreover, IsaA has been identified as a major antigen of S. aureus (30). Our RNA-seq results also revealed that the well-recognized virulence factor genes *slushABC* were strongly repressed in the absence of AgrA (Table S2). These three synergistic hemolytic peptides have shown their participation in pathogenicity (22, 31). Interestingly, the transcription of three of the seven genes of the *lug* operon (SLUG_08110, *lugB*, SLUG_08140), was slightly reduced in the Δ*agrA* mutant (Log_2_FC of -1.05 to -1.21). The downregulation of the other genes of the operon (*lugR*, *lugA*, *lugC*, and *lugD*) was also observed downregulated but with Log_2_ fold change from -0.43 to -0.9. Because these values were just below our threshold, these genes were not included in Table S2. *lug* encodes a complex allowing the non-ribosomal synthesis of lugdunin, a bioactive cyclic peptide that prevents S. aureus colonization of the human nose (32).

Our transcriptomic and proteomic results revealed that AgrA positively regulated the transcription of *asp23* in S. lugdunensis (Tables S1 and S2). In S. aureus, Asp23, belonging to the Gls24 superfamily has been identified as an alkaline shock protein whose corresponding gene expression is controlled by the alternative sigma factor SigB (33, 34). It may be suspected that *asp23* could be a virulence-associated gene because Gls24, first characterized as a general stress protein, has also been shown to be important for virulence in Enterococcus faecalis (35, 36).

### Regulatory networks.

It is evident that the 400 genes deregulated in the Δ*agrA* mutant of *S. lugdunensis* were not all directly under the control of AgrA. In this study, we observed that 20 transcriptional regulators exhibited a modification of their expression: nine were upregulated and 11 were repressed in the mutant strain (Table S2, Fig. 9). Most of them were suspected of being involved in cellular metabolism. However, interestingly, *saeR*, coding for the regulator of the two-component system (TCS) SaeRS, was upregulated in the Δ*agrA* mutant. In S. aureus, this TCS is an additional regulator of virulence factors *in vivo* (11, 37, 38).

## DISCUSSION

One of the major systems for regulating virulence in S. aureus is the *agr* locus. It upregulates secreted virulence factors and represses cell surface proteins in parallel with the regulation of the intracellular effector molecule RNAIII (5). It displays sequence homology and similar genetic organization to those of S. lugdunensis. Of note that in S. lugdunensis, the *hld* open reading frame coding for the delta-hemolysin was not found in the RNAIII sequence (23). In the present study, we intended to provide information on the role of the quorum-sensing regulator AgrA in this important opportunistic pathogen. Therefore, we constructed the Δ*agrA* mutant strain and performed phenotypic and global transcriptomic and proteomic studies.

Surprisingly, we observed that the Δ*agrA* mutant cells of S. lugdunensis showed reduced biofilm formation compared to the parental strain, contrary to what was observed for S. aureus and S. epidermidis (3, 39, 40). In staphylococci, several molecules have been shown to play an important role in biofilm formation: that is, the polysaccharide intracellular adhesin (encoded by the *ica* operon) (41–44) the biofilm associated protein (BAP) (45, 46), the accumulation associated protein (of S. epidermidis, homologous to SasG of S. aureus) (47–50) the major autolysin AtlE, and the enzyme for d-alanine esterification of teichoic acids DltA (51). No homologous proteins to BAP and SasG were found in S. lugdunensis N920143 and, based on our data, no altered expression of *icaABCD*, *dltABCD*, *atlE*, and *fbl* (encoding a fibrinogen binding protein) nor difference in production of the corresponding proteins were observed in the *ΔagrA* mutant compared to the parental strain. From the AgrA-regulated genes, it can be hypothesized that the downregulation of genes involved in iron homeostasis could lead to reduced biofilm formation. In S. lugdunensis, it has been clearly shown that the iron-limitation condition triggered biofilm production in parallel with overexpression of *isd* operon (16, 52). Note that the *isd* operon is not present in the S. epidermidis genome and was not identified as an AgrA-regulated gene in S. aureus (4).

The Agr system is necessary for the pathogenicity of staphylococci, and it was long believed that upregulation of virulence factors was mainly due to the RNAIII-dependent gene regulation (53). RNAIII acts as a post-translational regulator by interacting with target transcripts inhibiting the production of surface proteins (such as protein A) and other transcriptional regulators (53, 54). Interestingly, trans-complementation experiments showed that, despite the absence of delta-hemolysin and low overall homology with RNAIII from S. aureus, RNAIII from S. lugdunensis was able to regulate the expression of S. aureus target genes (23). We thus constructed the ΔRNAIII mutant and performed proteomic profiles comparison. Astonishingly, no significant difference in protein abundance was observed. Moreover, transcriptional analysis by RT-qPCR of some genes deregulated in the Δ*agrA* mutant did not reveal differential expression in the ΔRNAIII mutant either. Most virulence factors directly or indirectly under RNAIII control (enterotoxins, alpha-toxin, leukocidins, exoenzymes) in S. aureus are not present in S. lugdunensis, which makes the role of this regulatory RNA questionable in this strain. It is tempting to speculate that RNAIII from S. lugdunensis could be an evolutionary residue kept in the strain probably because it does not provide additional fitness cost.

In S. aureus, the AgrA transcriptional regulator binds to the promoters of the *agr* and RNAIII loci (P2 and P3) as well as the *psm* operons (9). The latter encodes phenol-soluble modulins (PSMs) which are peptide toxins (31). Likewise, in S. lugdunensis, we showed that the *agr* operon, RNAIII and *slushABC* (encoding PSMs) possessed a putative ArgA binding motif in the promoter regions and were downregulated in the Δ*agrA* mutant. The gene coding for IsaA was overexpressed in the Δ*agrA* mutant. IsaA is a soluble transglycosylase involved in peptidoglycan turnover and cell division but is highly immunogenic (29). Levels of anti-IsaA IgG were significantly higher in plasma samples from healthy S. aureus carriers than from non-carries (55). In this context, AgrA, repressing the expression of *isaA*, could participate in the immune escape of S. lugdunensis. AgrA has also been shown to be a positive regulator of Asp23, a stress protein that may be a virulence factor based on homology to Gls24 of E. faecalis (36). The *asp23* gene is well known to be regulated by the alternative sigma factor SigB and our result highlighted the likely cross talk between both transcriptional regulators AgrA and SigB (33).

Remarkably, AgrA regulated factors involved in colonization and stress responses. Lugdunin is a non-ribosomally synthesized bioactive peptide antibiotic produced by S. lugdunensis which prevents nasal colonization by S. aureus (32). In the present study, we showed that the expression of the *lug* operon was, at least in part, controlled by AgrA. Commensal staphylococci have to cope with a wide range of salt concentrations in human habitats. The osmotic stress response consists in the transport and accumulation of osmolytes such as glycine betaine, choline, L-proline, and taurine from the environment like skin or in the synthesis of betaine by choline-conversion pathway (27, 56). Under osmotic stress, the growth of the Δ*agrA* mutant was obviously more affected than the WT strain of S. lugdunensis. In parallel, we showed a highly reduced transcription of osmoprotectant transporter genes. This again proved that AgrA played an important role in the environmental adaptation of these bacteria.

As we also observed, the *agrA* mutants of S. aureus and S. epidermidis were more susceptible to hydrogen peroxide stress (10, 57). AgrA from S. aureus has a built-in oxidation-sensing mechanism *via* an intramolecular disulfide bond formation between Cys-199 and Cys-228 located in the DNA-binding domain of the regulator (10). This mechanism modulated transcription of *bsaA* coding for the glutathione peroxidase essential for the resistance to oxidative stress in S. aureus (10). S. lugdunensis possesses Cys-199 and Cys-228 amino acids in the AgrA sequence but no BsaA homologue. Nevertheless, our transcriptomic and proteomic data indicated that catalase, the major oxidoreductase enzyme crucial for H_2_O_2_ detoxification was less produced in the Δ*agrA* mutant. Several other AgrA upregulated genes products converged toward the protection to oxidative stress. Thus, the reduced transcriptional levels of *sodA* (superoxide dismutase), SLU_18820 (coenzyme A-disulfide reductase, CoADR), *crtM*, and *crtN* (dehydrosqualene synthase and dehydrosqualene desaturase, respectively) in the Δ*agrA* strain were in accordance with the lower resistance to H_2_O_2_ (in term of growth and survival) and the probably reduced production of the carotenoid pigment 4,4′-diaponeurosporene. In addition to its function to maintain the reduced state of Coenzyme A, CoADR was shown to be also important for S. aureus virulence (58, 59). Of note, *sodA*, *katA*, and SE0669 (coding for CoADR) were also found by microarray analysis of the Δ*agrA* mutant of S. epidermidis, and *crtN* was among *agr*-upregulated genes in S. aureus (4, 57). Both peptide methionine sulfoxide reductases (MsrA and MsrB) were less abundant in the Δ*agrA* mutant. These proteins are known to not only repair oxidative alterations to methionine residues through the oxidation/reduction cycle, but also serve as ROS scavengers and protect cells from more extensive damages (60). Oxidative stress is one of the main deleterious aggressions encountered by bacteria into phagocytic cells (60). By agar dilution method, using discs containing 1, 10, 100, and 1,000 µg of lysozyme, proteinase K, trypsin (trypsin 250) or pancreatic lipase (135 units/mg using olive oil at pH 7.7) as well as growth curves in the presence of 1 g/ml of these enzymes, we observed that both the Δ*agrA* mutant and the parental strains were resistant. Likewise, no difference of susceptibility to acidic pH of 5 was obtained (data not shown). Thus, the influence of AgrA to cope with oxidants appears to be closely related to the reduced ability of the Δ*agrA* mutant to survive in mice macrophages.

Approximately 16% of S. lugdunensis genes (400 over 2440 annotated genes) were deregulated in the Δ*agrA* mutant whereas 48 harbored putative AgrA binding sites in their promoter regions. In addition to the expected ArgA-box found in the intergenic region between the *agr* and RNAIII loci, it is noteworthy that this putative binding site was present in the promoter regions of *isdB*, *cspC*, *slushA*, and SLUG_20840 (coding an osmoprotectant transporter system). The important number of likely indirect AgrA-targets, mainly involved in diverse cellular metabolisms, may be explained by the impact on other regulators. AgrA controlled the expression of 20 transcriptional regulators among which the repression of the SaeRS TCS (11, 61). In S. aureus, the latter may be a virulence factor since influenced the transcription of hemolysins, leukotoxins, and coagulase genes, which are not present in *S. lugdunensis* (62). The role of the regulatory cascade involving AgrA and SaeRS remains to be elucidated. Contrary to S. epidermidis, neither *sarA* nor *sigB* transcription was influenced by the *agrA* deletion in S. lugdunensis. Nevertheless, some known S. aureus SigB regulated genes as *asp23*, *clpL*, *crtMN*, *opuD* and *katA* were members of the ArgA regulon in S. lugdunensis (63, 64). The alternative sigma B factor SigB of the RNA polymerase is a master regulator of a large regulon responding to a variety of stress conditions (63). Characterization of the SigB regulon in S. lugdunensis should provide additional information to better understand how this opportunistic pathogen can cope with environmental stresses.

In conclusion, S. lugdunensis cells with an active *agr* system appeared much well equipped to protect against environmental stresses (especially oxidative and osmotic), more able to form biofilm, and likely more virulent. Our phenotypic and global analyses proved that AgrA acted as a pleiotropic regulator and provided a snapshot of the direct and indirect targets. These data brought information about mechanisms that may at least in part explain how these commensal bacteria can become a worrying pathogen and thus support the use of AgrA as an interesting therapeutic target that can interfere with staphylococci pathogenicity (65).

## MATERIALS AND METHODS

### Bacterial strains and growth conditions.

The sequenced S. lugdunensis N920143 strain was used in this study (GenBank FR870271) (66). Overnight cultures of S. lugdunensis N920143 and Δ*agrA* mutant were diluted 2:100 in BHI. These bacterial suspensions were used to fill the wells of a 96-wells flat-bottom sterile polystyrene microplate. Growth measurements (OD_600_ every 10 min) were performed using the microtiter plate reader Tecan infinite 200 pro (Tecan, Männedorf, Switzerland) and *P values* were determined using a variance Student's *t* test.

Bacterial colony pigmentation was macroscopically detected after 24 h of incubation at 37°C on agar TS and BCP agar.

### Construction of the S. lugdunensis N920143 Δ*agrA* mutant and complemented strains.

Isolates were grown overnight at 37°C on BHI agar. DNA was extracted using the InstaGene Matrix kit (Bio-Rad, Hercule, CA, USA) according to the manufacturer’s instructions. PCRs were performed using a BIO-RAD Thermal Cycler (Bio-Rad) in a final volume of 50 μl containing 0.5 μl Q5 polymerase (New England BioLabs, Every, France), 10 μM each primer, and 2 μl of DNA. When necessary, PCR products were purified and verified by sequencing. The plasmids were extracted with QIAprep Spin Miniprep Kit (Qiagen, Hilden, Germany).

The *agrA* deletion mutant was constructed by homologous recombination using pMAD plasmid (67). The fragment containing the upstream region of *agrA* (with respect to the *agrA* translation initiation site) and the downstream region (with respect to the *agrA* stop codon) were amplified by a two-step overlap PCR using the oligonucleotide pairs AgrA-1F-EcoRI/AgrA-1R-NotI and AgrA-2F-NotI/AgrA-2R-BamHI (Table S4). The purified PCR products were digested with NotI restriction enzyme (New England BioLabs) and ligated into the pTOPO plasmid with TOPO TA Cloning Kit (ThermoFisher, Carlsbad, USA) and cloned into Escherichia coli TOP10. PCR using the oligonucleotide M13-F and M13-R was performed for verification (Table S4). The purified PCR product was then digested with EcoRI restriction enzyme and ligated into the pMAD vector with T4 DNA ligase (New England BioLabs) following the manufacturer’s instructions and then cloned into E. coli SL01B (68). Clones containing the resulting recombinant pMADΔ*agrA* vector were selected on BHI supplemented with ampicillin (50 μg/ml). pMADΔ*agrA* was purified and used to transform the WT S. lugdunensis N920143 strain by electroporation as previously described (68). After incubation at 30°C for 5 days, clones containing the plasmid integrated into the chromosome were selected, and the double-crossover event was performed by a shift of temperature to 43°C and in the presence of erythromycin (67). The loss of the free plasmid was obtained after successive shifts between 30 and 43°C and in the absence of antibiotics. Successful deletion of the *agrA* gene was verified by PCR and sequencing.

The RNAIII deletion mutant was also constructed by homologous recombination using pMAD plasmid and oligonucleotide pairs listed in Table S4 using the protocol described above.

For the complemented strain, the entire *agr* operon, obtained by PCR amplification using Agr-comp-pRB473-F and Agr-comp-pRB473-R primers (Table S4), was clone into plasmid pRB473 after restriction by SmaI (69). As previously described, recombinant plasmid was used to first transform E. coli SL01B. PCR using the oligonucleotides pRB473-F and pRB473-R was performed for verification (Table S4). This construction as well as the native plasmid were then introduced in competent cells of Δ*agrA* mutant and selected on agar plates containing 30 µg/ml of chloramphenicol.

### Biofilm formation.

To generate biofilms, 50 μl of overnight culture (adjusted at OD_600_ of 0.1 with BHI) of *S. lugdunensis* N920143 and Δ*agrA* mutant were inoculated into the wells of the μ-Plate Angiogenesis 96 well (Ibidi, Madison, WI, USA). After 20 h, the planktonic cells were discarded, and the wells were washed with 50 μl of sterile water. Then, 25 μl of labeling solution (LIVE/DEAD™ BacLight™ Bacterial Viability Kit, Invitrogen) was added. Following incubation for 15 min at 20–25°C, the biofilms were mounted using one drop of ProLong Diamond Antifade Mountant (Life Technologies, OR, USA), and the plate was incubated for 24 h at room temperature in darkness. The live and dead fluorescent cells present in the biofilms were observed using the inverted microscope Nikon Eclipse Ti-S (x60 microscopic lens) equipped with the Nikon Digital Sight DS-Ri1 camera (Nikon, Tokyo, Japan). The pictures were obtained and analyzed using the NIS Element AR software (version 5.01.00) (Nikon). The results represent the mean of biofilm volume (μm^3^) of three independent experimental values, and *P values* were determined using a variance Student's *t* test.

Biofilm formation was also detected using the crystal violet staining method previously described (70). Overnight cultures of S. lugdunensis N920143 and Δ*agrA* mutant were diluted to obtain OD_600_ of 0.1 in BHI. Sterile polypropylene microplates (Eppendorf, Montesson, France) were loaded with 200 μl of bacterial suspensions and incubated for 24 h at 37°C. Adherent cells were stained with 0.1% crystal violet for 15 min and, after three washes with sterile water, wells were air-dried. For quantitative estimation of the biofilm density, bound crystal violet was solubilized with 70% ethanol and the absorbance of the solubilized dye was read at 600 nm with the microtiter plate reader. Three independent experiments (each in duplicate) were performed, and *P values* were determined using a two-tailed, two-sample unequal variance Student's *t* test.

### Survival experiment in mice peritoneal macrophages.

Survival of S. lugdunensis N920143 and Δ*agrA* mutant in mice peritoneal macrophages was tested using an *in vivo–in vitro* infection model, as previously described (71). Bacterial cells were pelleted and suspended in an adequate volume of phosphate-buffered saline (PBS) for injection. Male BALB/c mice (10 weeks old; Harlan Italy S.r.l., San Pietro al Natisone, Udine, Italy) were infected with 4.8 × 10^7^ ± 0.3 × 10^7^ cells of each strain (estimated by CFU determination) by intraperitoneal injection of 200 μl of the PBS-staphylococcus suspension. After a 6-h infection period, the macrophages were collected by peritoneal lavage, centrifuged, and suspended in Dulbecco's modified Eagle's medium (DMEM) containing 10 mM HEPES, 2 mM glutamine, 10% fetal bovine serum, and 1X nonessential amino acids, supplemented with vancomycin (10 μg/ml) and gentamicin (150 μg/ml). The cell suspension was dispensed into 24-well tissue culture plates and incubated at 37°C under 5% CO_2_ for 2 h. After exposure to antibiotics (i.e., 8 h postinfection) to kill extracellular bacteria, the infected macrophages were washed, and triplicate wells of macrophages were lysed with detergent. After dilution with BHI broth, the lysates were plated on BHI agar to quantitate the viable intracellular bacteria. The remaining wells of infected macrophages were maintained in DMEM with the antibiotics for the duration of the experiment. The same procedure was performed at 24, 48, and 72 h postinfection. In parallel, supernatant fluids from each well were removed, and extracellular bacteria were quantitated by counting on BHI agar plates. To assess their viability, macrophages were detached from tissue culture wells with cell scrapers and stained with trypan blue, and viable macrophages were counted with a hemacytometer. The procedure was repeated three times, and the results were analyzed using a one-way analysis of variance with a Bonferroni correction posttest with SPSS statistical software (SPSS, Chicago, IL, USA). All statistical analyses were performed using Prism software (version 5.00) for Windows (GraphPad Software, San Diego, CA, USA). For all comparisons, a *P value* of less than 0.05 was considered significant.

The experiments were performed under a protocol approved by the Institutional Animal Use and Care Committee at Università Cattolica del S. Cuore, Rome, Italy (Permit number: Z21, 11/01/2010) and authorized by the Italian Ministry of Health, according to Legislative Decree 116/92, which implemented the European Directive 86/609/EEC on laboratory animal protection in Italy. Animal welfare was routinely checked by veterinarians of the Service for Animal Welfare.

### Osmotic and oxidative stress conditions.

Overnight cultures of S. lugdunensis N920143 and Δ*agrA* mutant were diluted 2:100 in BHI with 10% NaCl or 0.4 mM H_2_O_2_. These bacterial suspensions were used to fill the wells of a 96-wells flat-bottom sterile polystyrene microplate and growth measurements (OD_600_ every 10 min) were carried out using the microtiter plate reader. Experiences were performed at least three times and *P values* were determined using a variance Student's *t* test.

The oxidative killing assays of S. lugdunensis N920143 and Δ*agrA* mutant were performed as previously described (16). Briefly, bacteria were grown 24 h in BHI broth and subcultured in 10 ml broth at a starting density of OD_600_ of 0.1. Cultures were grown to mid-exponential phase (OD_600_ = 0.5) and 1 mM H_2_O_2_ was added. Samples were immediately taken, as well as 1 h and 2 h following H_2_O_2_ challenge at 37°C, and rapidly diluted in 0.9% NaCl solution. Viability was determined by spreading of appropriate serial dilutions on BHI agar, and CFU were determined after 48 h of incubation at 37°C.

### Mass-spectrometry.

The total protein extraction and the mass-spectrometry were done as described previously (16). Briefly, 200 ml of S. lugdunensis N920143 and Δ*agrA* mutant cell cultures were centrifuged (6000 g) for 15 min at 4°C, washed twice with recovery buffer (Tris HCl 50 mM, Na_2_SO_4_ 50 mM, glycerol 15%), and incubated for 12 h at −80°C. Cells were disrupted using the Fast Prep instrument (MP Biomedical LLC, Santa Ana, CA, USA) for 3 min at 6.5 m/s followed by centrifugation for 10 min at 10,000 g at 4°C to remove the cell debris. Proteins were prepared from three independent biological samples and the quantification was realized with the Pierce BCA protein assay kit (ThermoFisher, Waltham, MA, USA).

Five μg of each protein extract were digested with trypsin/Lys-C overnight at 37°C. After protein or peptide desalting and concentration, the chromatography step was performed on a NanoElute (Bruker Daltonics, Billerica, MA, USA) ultrahigh-pressure Nano flow chromatography system. Mass spectrometry experiments were carried out on a TIMS-TOF pro mass spectrometer (Bruker Daltonics) and spectra were acquired in the positive mode in the mass range from 100 to 1700 *m/z*. These data were processed with MaxQuant version 1.6.7.0 and the mass spectrometry (MS)/MS spectra were searched by the Andromeda search engine against the Uniprot S. lugdunensis database. Bioinformatic analysis and visualization were performed in Perseus. Three sample tests were performed using Student's *t* test with a Permutation-based FDR of 0.05.

### RNA extraction.

RNAs were extracted from cells of *S. lugdunensis* N920143 and Δ*agrA* mutant cultured in BHI harvested in the late-exponential phase (OD_600_ of 1) as previously described (16). Following incubation of the cell pellets for 12 h at −80°C, RNAs were extracted with the Direct-zol RNA miniprep kit (Zymo Research, Irvine, CA, USA) and residual DNA was removed using Turbo DNase (ThermoFisher) according to the manufacturer’s instructions. RNAs integrities and quantifications were measured by the Agilent TapeStation system (Agilent, Santa Clara, CA, USA). Four independent samples for each strain were used.

### RNA-Seq and reads mapping.

cDNAs from the RNA samples were sequenced with the Illumina HiSeq 4000 platform generating reads of 50 pb (iGE3 Genomics Platform, University of Geneva, Swiss). For each condition, RNA-Seq analyses have been carried out using the four independent RNA samples. The fastqc software, the STAR algorithm (version 2.7.5b), and the package HTSeq (version 0.13.5) were used to perform the quality assessments, reads mapping to the S. lugdunensis genome (assembly: ASM27046v1 accession number: NC_017353.1), and table of counts, respectively. For the identification of genes differentially expressed in the Δ*agrA* mutant versus the S. lugdunensis N920143 parental strain, normalized data of reads were compared using the R package edgeR (version 3.26.8) and correction of the *P value* was performed by the Benjamini–Hochberg method. Values of log_2_ fold change (Log_2_FC) less than −1 or higher than 1 with a corrected *P value* less than 0.05 were considered to be statistically down or up deregulated.

### RT-qPCR experiments.

One μg of RNA was reverse transcribed using the BIORAD iScript Select cDNA synthesis Kit (Bio-Rad). The resulting cDNA was then amplified by qPCR using iSC SYBR green supermix in the iCycler iQ instrument (Bio-Rad) according to the manufacturer’s instructions. The primers used for this study are listed in Table S4. These experiments were performed in triplicate from three independent RNA extractions. We considered a significant modification in gene expression when the fold changes were greater than 2 or less than -2 with a *P value* < 0.05.

### Search for the putative AgrA binding site.

The reference genome used for this analysis was ASM27046v1 (https://www.ncbi.nlm.nih.gov/assembly/GCF_000270465.1). First, using a homemade script, the coordinates of the non-coding regions and all the CDS were extracted. Then the coordinates of the putative AgrA-binding motif defined as “WMMGTTNRK” (8) were extracted from the fasta file of the genome using SeqUtils and SeqIO from Biopython, on both DNA strands. Finally, among genes whose transcriptions were modified in the Δ*agrA* mutant, those with a motif found in the non-coding regions corresponding to the putative promoter regions, were suspected to be directly under AgrA control.

### Data availability.

The LC-MS/MS proteomics data have been deposited to the ProteomeXchange Consortium via the PRIDE partner repository with the data set identifier PXD028347 (https://www.ebi.ac.uk/pride).

All the RNA-Seq data are available in the GEO database with accession number GSE181857 (https://www.ncbi.nlm.nih.gov/gds/).
