# Supplementary material for: Inactivation of the Response Regulator AgrA Has a Pleiotropic Effect on Biofilm Formation, Pathogenesis and Stress Response in Staphylococcus lugdunensis
Source: Microbiol Spectr. 2022 Feb 9;10(1):e01598-21. doi: 10.1128/spectrum.01598-21 (PMC8826819; doi:10.1128/spectrum.01598-21)
Supplement: SUPPLEMENTAL FILE 4 — Supplemental material. Download SPECTRUM01598-21_Supp_4_seq17.pdf, PDF file, 0.7 MB [file spectrum01598-21_supp_4_seq17.pdf]

SUPPLEMENTAL MATERIAL

LEGENDS

TABLE S1 List of proteins significantly upregulated and downregulated of growing cell (OD_{600} of 1) of *S. lugdunensis* N920143 compared to Δ*agrA* mutant.

TABLE S2 List of all genes differentially expressed in *S. lugdunensis* N920143 cells compared to Δ*agrA* mutant cells (OD_{600} of 1).

TABLE S3 Summary of RNAseq results of the samples of *S. lugdunensis* N920143 and Δ*agrA* mutant.

TABLE S4 List of oligonucleotides used in this study.

FIG S1 Complementation experiments. **A** growth curves in BHI with 10% NaCl of *S. lugdunensis* Δ*agrA* mutant containing the empty plasmid pRB473 (continuous line), and Δ*agrA* mutant with the plasmid pRB473-*agr* (complemented strain) (dotted line). **B** growth curves in BHI with 0.4 mM H_2O_2 of *S. lugdunensis* Δ*agrA* containing the empty plasmid pRB473 (continuous line), and Δ*agrA* mutant with the plasmid pRB473-*agr* (complemented strain) (dotted line). **C** Transcriptional changes (Log_2FC) of RNAIII, *crtM*, *crtN*, *slushABC*, and *SLUG_07290* in the Δ*agrA* mutant compared to the wild type strain (black bars) and in complemented strain (Δ*agrA* mutant with the plasmid pRB473-*agr*) compared to the Δ*agrA* strain (white bars). Asterisks indicate p values < 0.05.

FIG S2 Volcano plots of proteins differentially expressed of growing cell (OD_{600} of 1) of *S. lugdunensis* N920143 compared to Δ*agrA* mutant.

FIG S3 Volcano plots of genes differentially expressed of growing cell (OD_{600} of 1) of *S. lugdunensis* N920143 compared to Δ*agrA* mutant.

TABLE S3 Summary of RNAseq results of the samples of *S. lugdunensis* N920143 and Δ*agrA* mutant.

	***S. lugdunensis* N920143 wild type**				***S. lugdunensis* N920143 *agr* deleted**			
	1	2	3	4	1	2	3	4
**Overall results**								
Number of input reads	29827506	33177543	34470980	31888778	32495196	31285384	29215079	31391569
Average input read length	50	50	50	50	50	50	50	50
Mean Quality	39.8	39.81	39.83	39.8	39.42	39.43	39.40	39.44
(%) > Q30	98.46	98.5	98.52	98.48	99.57	99.61	99.56	99.60
**Unique reads**								
Uniquely mapped reads number (%)	28976728 (97.15 %)	32320911 (97.42 %)	33762650 (97.95 %)	31205204 (97.86 %)	31426765 (96.71 %)	30426886 (97.26 %)	28239556 (96.66 %)	30783448 (98.06 %)
Average mapped length	49.94	49.94	49.94	49.94	49.94	49.95	49.94	49.95
**Multi-mapping reads**								
Number of reads mapped to multiple loci (%)	594203 (1.99 %)	492013 (1.48 %)	410947 (1.19 %)	518997 (1.63 %)	968870 (2.98 %)	550622 (1.76 %)	648812 (2.22 %)	485314 (1,55 %)
Number of reads mapped to too many loci	31	22	12	14	24	11	17	9
**Unmapped reads**								
Number of reads unmapped: too many mismatches	0	0	0	0	0	0	0	0
Number of reads unmapped: too short (%)	256245 (0.86 %)	364066 (1.10 %)	297024 (0.86 %)	164342 (0.52 %)	99284 (0.31 %)	307564 (0.98 %)	326342 (1.12 %)	122274 (0.39 %)
Number of reads unmapped: other	299	531	347	221	253	301	353	524

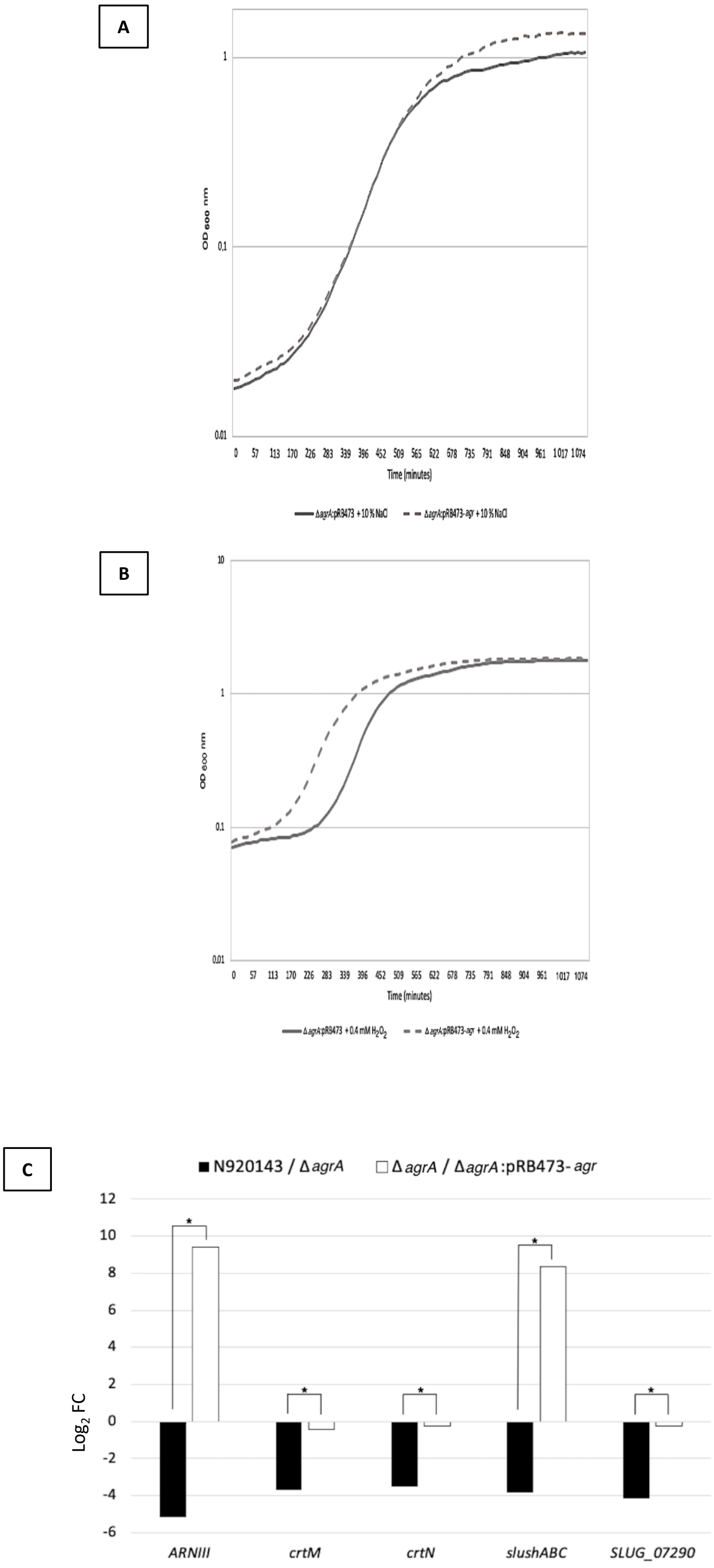

A
OD 600 nm
1
0.1
0.01
0 57 113 170 226 283 339 396 452 509 565 622 678 735 791 848 904 961 1017 1074
Time (minutes)
ΔagrA:pRB473 + 10 % NaCl
ΔagrA:pRB473-agr + 10 % NaCl
B
OD 600 nm
10
1
0.1
0.01
0 57 113 170 226 283 339 396 452 509 565 622 678 735 791 848 904 961 1017 1074
Time (minutes)
ΔagrA:pRB473 + 0.4 mM H_2O_2
ΔagrA:pRB473-agr + 0.4 mM H_2O_2
C
N920143 / ΔagrA
ΔagrA / ΔagrA:pRB473-agr
Log_2 FC
12
10
8
6
4
2
0
-2
-4
-6
*
ARNIII
crtM
crtN
slushABC
SLUG_07290

FIG S1

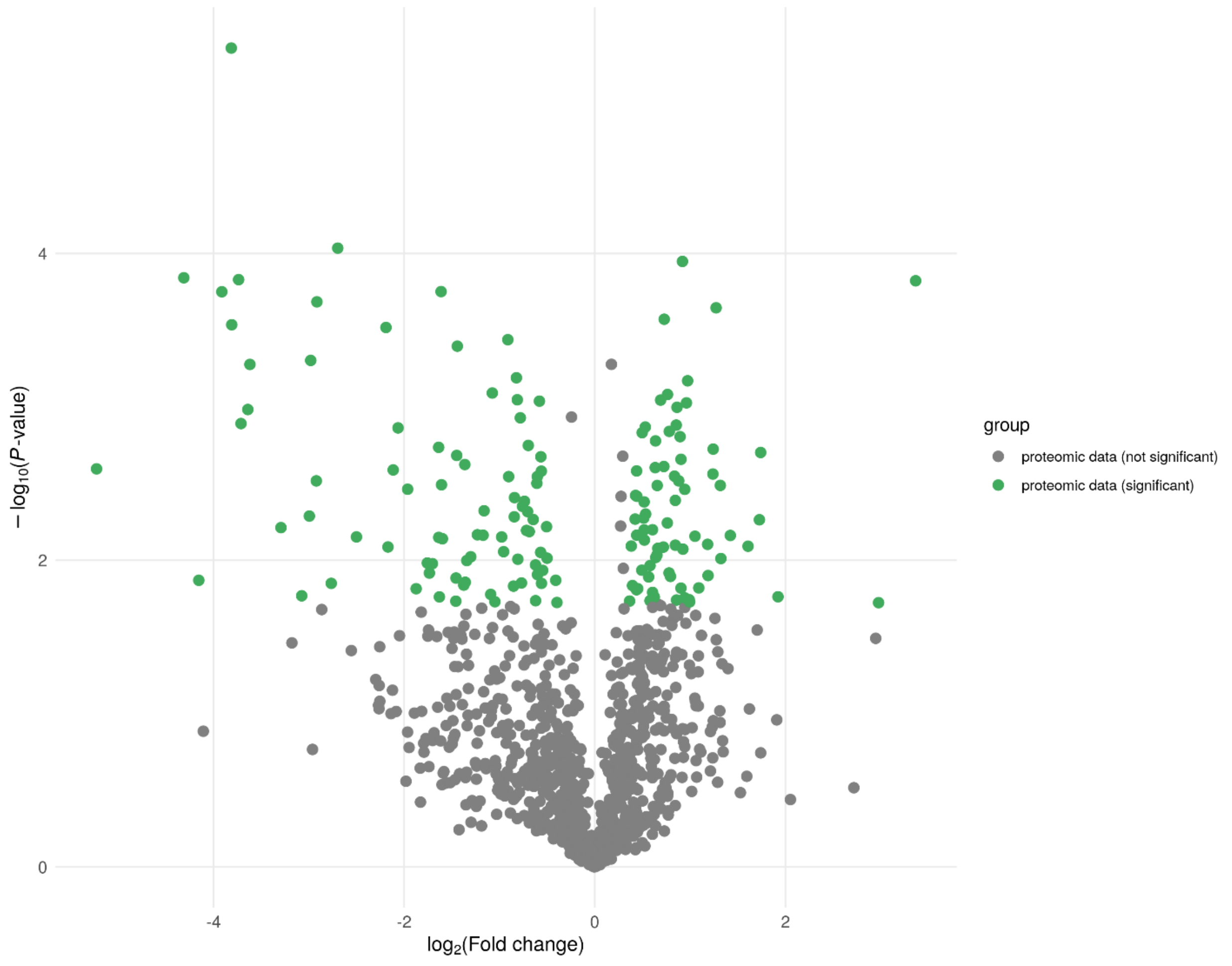

4
2
0
-4
-2
0
2
$-\log_{10}(P\text{-value})$
$\log_2$(Fold change)
group
proteomic data (not significant)
proteomic data (significant)

FIG S2

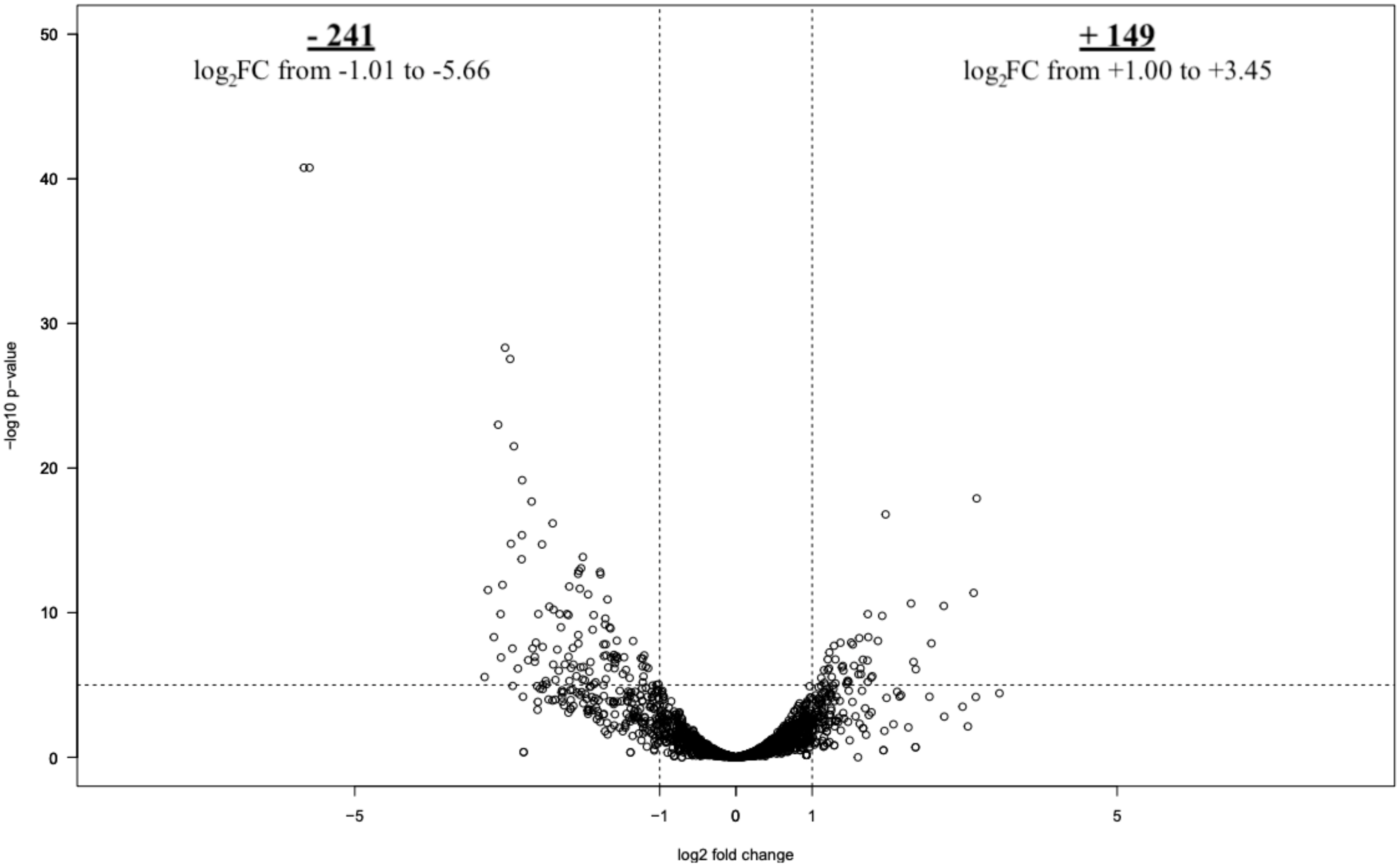

- 241
log_2FC from -1.01 to -5.66
+ 149
log_2FC from +1.00 to +3.45
50
40
30
20
10
0
−log10 p−value
−5
−1
0
1
5
log2 fold change

FIG S3
